# Genome-Wide Identification and Analysis of the Chicken Basic Helix-Loop-Helix Factors

**DOI:** 10.1155/2010/682095

**Published:** 2010-05-03

**Authors:** Wu-yi Liu, Chun-jiang Zhao

**Affiliations:** ^1^Department of Animal Science, China Agricultural University, Yuanmingyuan West Road No.2, Beijing 100093, China; ^2^Department of Biology Science, Fuyang Normal College, Qinghe East Road No.741, Fuyang 236041, China

## Abstract

Members of the basic helix-loop-helix (bHLH) family of transcription factors play important roles in a wide range of developmental processes. In this study, we conducted a genome-wide survey using the chicken (*Gallus gallus*) genomic database, and identified 104 bHLH sequences belonging to 42 gene families in an effort to characterize the chicken bHLH transcription factor family. Phylogenetic analyses revealed that chicken has 50, 21, 15, 4, 8, and 3 bHLH members in groups A, B, C, D, E, and F, respectively, while three members belonging to none of these groups were classified as ‘‘orphans”. A comparison between chicken and human bHLH repertoires suggested that both organisms have a number of lineage-specific bHLH members in the proteomes. Chromosome distribution patterns and phylogenetic analyses strongly suggest that the bHLH members should have arisen through gene duplication at an early date. Gene Ontology (GO) enrichment statistics showed 51 top GO annotations of biological processes counted in the frequency. The present study deepens our understanding of the chicken bHLH transcription factor family and provides much useful information for further studies using chicken as a model system.

## 1. Introduction

Transcription factors of the basic helix-loop-helix (bHLH) family play important roles in regulation of cell proliferation and differentiation, cell lineage determination, myogenesis, neurogenesis, hematopoiesis, sex determination, gut development, as well as other essential processes in organisms ranging from yeast to mammals [1–3]. The first characterization of bHLH transcription factors was reported on the murine factors E12 and E47 [4]. In 1997, a large scale phylogenetic analysis based on 122 bHLH sequences leaded to a natural classification of different bHLH transcription factors into four monophyletic protein groups named A, B, C, and D in an attempt to functionally segregate bHLH proteins [1]. Since then, numerous bHLH proteins have been identified in animals, plants, and fungi. In phylogenetic analyses of over 400 bHLH proteins, Ledent et al. had defined 45 orthologous families and six higher-order groups for all the identified bHLH proteins, and the families were named after the first discovered or best-known member [1, 3, 5]. In brief, Groups A and B bHLH proteins bind to core DNA sequences typical of E boxes (CANNTG), in which group A binds to CACCTG or CAGCTG and group B binds to CACGTG or CATGTTG. Group C proteins are complex molecules with one or two PAS domains following the bHLH motif. They bind the core sequence of ACGTG or GCGTG. Group D proteins lack a basic domain and form inactive heterodimers with group A proteins. Group E proteins bind preferentially to sequences typical of N boxes (CACGCG or CACGAG). They usually contain two additional domains named “Orange” and “WRPW” peptide in their carboxyl terminus. Group F proteins have the COE domain which has an additional domain involved in both dimerization and DNA binding.

BHLH transcription factors share a common bHLH structural motif or domain of approximately 60 amino acids which contains a basic region and two helices separated by a loop (HLH) region of variable length [2, 3]. The basic region works as a DNA-binding domain. The amphipathic *α*-helices of two bHLH proteins can interact, and the HLH domain promotes dimerization, allowing the formation of homodimeric or heterodimeric protein complexes between different members [3]. Atchley et al. developed a predictive motif for the bHLH domains based on 242 bHLH proteins, in which 19 conserved sites were found within the bHLH domain [6]. Atchley et al. showed that a sequence with less than 8 mismatches to the predictive motif was possibly a bHLH protein [6], and later other researchers found that a sequence with even 9 mismatches could also be a potential bHLH protein [7]. 

Given the importance of the bHLH genes in development, it would be desirable to have a more refined classification scheme of the various types of bHLH motifs, as well as a better understanding of their evolutionary relationships both within and between organisms. Recently, a growing number of bHLH genes have been identified, and bHLH transcription factor families have been analyzed in many organisms whose genomes have been sequenced [5, 8–11]. However, the family of bHLH transcription factors has not been comprehensively studied and characterized in chicken. A preliminary identification of 104 bHLH proteins was reported in a study of zebrafish bHLH transcription factors [9], in which fifteen were EST (expressed sequence tag) sequences without special annotation. However, the chicken bHLH proteins were not analyzed in detail and many potential bHLH members were missed in their study. An initial BLAST search performed by our lab identified more than 150 bHLH members, suggesting great diversity in this genetic family that would justify a complete genomic survey of basic helix-loop-helix transcription factors in chicken. 

The chicken (*Gallus gallus*) is both a global food source and a model organism for biology researches. The draft genome sequence of the red jungle fowl, *Gallus gallus*, and those of three domestic chicken breeds (a broiler, a layer and a Chinese silkie) has been completed [12, 13], and the latest version of chicken genome assembly (build 2.1) has been available on GenBank since November 21 2006. In this study, we used the criteria developed by Atchley et al. [6] and the 45 representative bHLH domains defined by Ledent et al. [5] to Blast-search the chicken genomic databases and finally identified 104 *Gallus gallus* bHLH (GgbHLH) sequences. We next made phylogenetic analyses of the chicken bHLH family using 118 human bHLH domains, allowing us to define the chicken bHLH “subfamilies”. We also compared the bHLH families in a few vertebrate and invertebrate species and analyzed the enriched Gene Ontology (GO) terms for the chicken bHLH transcription factors.

## 2. Materials and Methods

### 2.1. Identification of Protein Sequence, Genomic Contig, and Chromosome Location

We initially followed the criteria developed by Atchley et al. [6] to define a bHLH protein, and retrieved 7 chicken bHLH sequences in primary searches based on the consensus sequences predicted by Atchley et al. based on 242 sequences for bHLH domains (mRNA accession number: AJ579995.2, AJ579996.2, D90157.1, D10599.1, NM_204679.1, NM_204214.1, and NM_001030363.1). The predictive motif is “++*X*_(3–6)_ *E*+*X* *R* *X*_(3)_ *α* *N* *X*_(2)_Φ*X*_(2)_ *L*+*X*_(5–22)_+*X*_(2)_ *K* *X*_(2)_ *σ* *L* *X*_(2)_ *A* *σ* *X* *Y* *α* *X*_(2)_ *L*”. Where + = *K*, *R*; *α* = *I*, *L*, *V*; Φ = *F*, *I*, *L*; *δ*= *I*, *V*, *T*; *E*, *R*, *K*, *A*, and *Y* are as defined; *X* = any residue; *X*_(i)_ = any *i *residues; and *X*_(*i*–*j*)_ = *i* to *j* of any residues. 

The 7 primer sequences and those 45 representative bHLH domains from the tables of Ledent et al. [5] were used to make genomewide TBLASTN and BLASTP searches of the chicken bHLH domains. Each sequence was used to perform searches against the chicken protein and genomic databases of NCBI, including RefSeq protein, RefSeq RNA, *Ab initio* protein, Build protein, Build RNA, and Non-RefSeq protein (http://www.ncbi.nlm.nih.gov/genome/seq/BlastGen/BlastGen.cgi?taxid=9031). Stringency was set to *E *< 10 in order to obtain all bHLH-related sequences for later examination. With TBLASTN against the chicken databases, we obtained all putative bHLH proteins that had more than 10 conserved amino acids among the 19 residues [7]. Each sequence was used to perform a second TBLASTN and PSI-BLAST (position specific iterative BLAST) searches against the chicken genomic databases. This procedure was repeated three times. Subsequently, redundant sequences of candidate bHLH proteins or genes were removed according to their corresponding sequencing bacterial artificial chromosome clone (genome contig) serial numbers, gene ID, protein ID, coding regions, and sequence alignments. The subject sequences obtained were manually examined to find introns within the bHLH motifs using the NetGene2 online (http://www.cbs.dtu.dk/services/NetGene2/). Protein sequence accession numbers were obtained by using the amino acid sequence of each identified chicken bHLH motif to conduct BLASTP searches of all the chicken protein databases. Genomic contig numbers were obtained by using the amino acid sequences of each identified chicken bHLH motif to conduct a TBLASTN search of the chicken genome sequence assembly of “reference only”. Both searches above used 0.01 as their *E* value and were not filtered. The chromosome location of each identified chicken bHLH sequence was obtained by searching against the chicken genome view project (http://www.ncbi.nlm.nih.gov/projects/mapview/map_search.cgi?taxid=9031).

### 2.2. Sequence Alignment and Motif Comparing

All sequences that passed the examination above were aligned using ClustalX 2.0 [16] with default settings. The aligned bHLH domains were shaded using GeneDoc 2.6.02 [17] and copied into a RTF file for further annotation. Sequences were compared according to conserved amino acid numbers.

### 2.3. Phylogenetic Analysis and Testing for Positive Selection

Phylogenetic analyses were conducted using MRBAYES 3.1.2 [18, 19] and PHYML 2.4.4 [20].The obtained GgbHLH sequenceswere used to construct phylogenetic trees of Bayesian inference and maximum likelihood matching with the 118 human bHLH domains [5]. Initial alignments were generated using ClustalX to prepare phylip format files. Maximum likelihood (ML) analyses were performed using the Jones-Taylor-Thornton (JTT) amino-acid substitution model [21], the frequencies of amino acids being estimated from the data set, and rate heterogeneity across sites being modeled by two rate categories (one constant and eight *γ*-rates). Statistical support for the different internal branches was assessed by bootstrap resampling with 100 replicates in PHYML [20]. Bayesian inference was performed with MRBAYES [18, 19]. We used the JTT substitution frequency matrix [21] with among-sites rate variation modeled by a discrete *γ* distribution with four equally probable categories. Two independent Markov chains were run, each containing from 100,000 to 14,000,000 Monte Carlo steps until the standard deviation of split frequencies was below 0.01. Trees were saved every 100 generations. The trees obtained in the two runs of Markov chains were meshed and the first 25% of the trees were discarded as “burnin”, and only the 50% majority consensus trees were displayed. All trees were edited by means of MEGA 4.0 [22].

### 2.4. Gene Ontology (GO) Distribution and Enrichment Analysis

The Gene Ontology (GO) hierarchy annotations were downloaded from the Gene Ontology database (http://omicslab.genetics.ac.cn/GOEAST/index.php). Enrichment for GO categories was also analyzed using the toolkit GOEAST [15] which reports enrichment (including a hyper-geometric *P* value), with respect to GO categories.

## 3. Results and Discussion

### 3.1. Chicken bHLH Proteins

TBLASTN and BLASTP searches with the 7 chicken bHLH primers and the 45 representative bHLH domains initially identified 151 sequences, and the followed manual improvement and examination resulted in the identification of 104 *Gallus gallus* bHLH (GgbHLH) proteins (listed in Table 1). The number is equivalent to but more accurate than previous searches in the zebrafish study [10]. Most of the bHLH domains we obtained had more than 10 conserved amino acids among the 19 residues [7].

**Figure 1:**
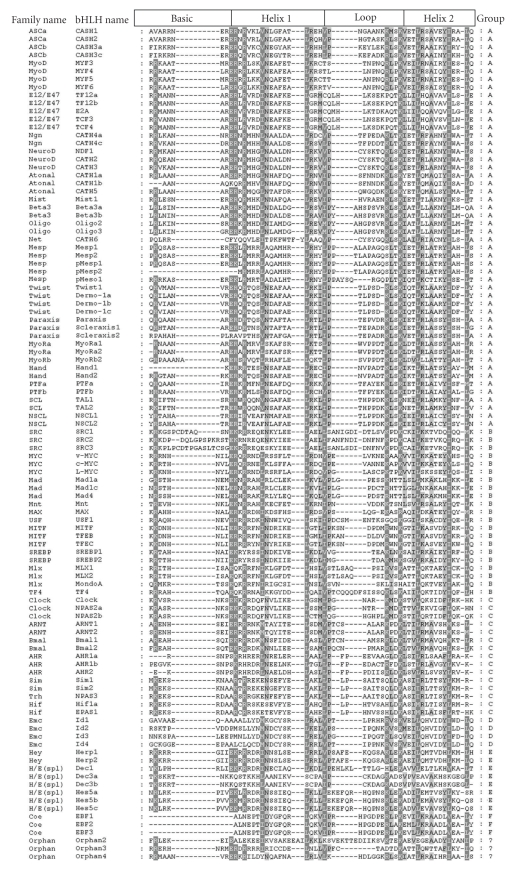
Alignment of the 104 chicken bHLH protein domains shaded using Genedoc. Designation of basic, helix 1, loop and helix 2 follows [1], and Ferre-D et al. [14]. Detailed information of the 104 chicken bHLH proteins was attached in Table 1.

The names of the 104 chicken bHLH proteins are listed in Table 1. Each chicken bHLH protein was named according to its phylogenetic relationship with the corresponding human homologue(s). Where one human bHLH sequence has two or more chicken homologues, we used “a”, “b”, and “c”, or “1”, “2”, and “3”, and so forth, to number them. For instances, two homologues of the human gene Mlx were found in chicken. Thus, the chicken genes were named Mlx1 and Mlx2, respectively. It was found that chicken has 50, 21, 15, 4, 8, and 3 bHLH members in groups A, B, C, D, E, and F, respectively. Members of three families, for example, Delilah, Fig*α*, and AP4 were not found in the chicken proteome databases. Three members could not be assigned to any known families and were classed as “orphans”. It should be noticed that, among the 104 chicken bHLH proteins, the expression of 29 hypothetical protein and/or predicted proteins such as LOC768612 was confirmed with corresponding EST sequences(Supplemental Table 1). Alignment of all the 104 chicken bHLH domains is shown in Figure 1.

It was found that chicken and human each possess unique bHLH genes. For instance, chicken homologues were not found for human Hath4b, NDF2, Oligo1, MyoRb1, L-Myc2, Mad1b, Lyl1, Fig*α*, Mxi1, Mnt, USF2, USF3, TFE3, AP4, TF4, Hif3*α*, NPAS1, HEYL, Hey4, Hes1, Hes2, Hes3, Hes4, Hes6, Hes7, EBF4, Orphan1, Orphan2, and Orphan4 genes. On the contrary, chicken either has extra members in certain bHLH families or has multiple homologues corresponding to one specific human bHLH sequence. The former includes TF12b, CATH1b, Scleraxis2, Mad1c, NPAS2b, and AHR1b. The latter includes Mesp1 and Mesp2, pMesp1, and pMesp2 (homologues of human pMesp1); Dermo-1a, Dermo-1b, and Dermo-1c (homologues of human Twist2); Hes5a, Hes5b, and Hes5c (homologues of human Hes5) (Table 1).

### 3.2. Phylogenetic Analyses and Identification of Orthologous Families

Classification of human bHLH family members has been extensively studied [5, 9, 10]. Thus, human bHLH members can be used as a good reference for homologue identification of bHLH members in other organisms. Although orthologue identification has been accompanied by much uncertainty since there is no absolute criterion that can be used to decide whether two genes are orthologous [3], by constructing phylogenetic trees using robust methods and setting an adequate standard for bootstrap values, phylogenetic analysis has remained an effective measure for homologue identification [9]. Herein, phylogenetic analyses of Bayesian inference (BI) and maximum likelihood estimate (ML) were used to identify unknown bHLH sequences in different phylogenetic trees with other known bHLH members. If the unknown sequence forms a monophyletic clade with a known bHLH member or family with bootstrap value is >50 in phylogenetic trees, the known member will be regarded as a homologue of the unknown sequence. 

In this study, the phylogenetic analyses with the known 118 HsbHLH domains revealed that the 104 GgbHLH belong to 42 subfamilies with the phylogenetic trees of Bayesian inference and maximum likelihood estimate. The bootstrap values obtained that support the formation of a monophyletic clade with its human homologue are listed in Table 1. Table 1 indicates that the bootstrap support of Bayesian inference was robust enough for identifying chicken bHLH sequences as homologues of specific human bHLH members, but that of maximum likelihood estimate varied greatly. The topologies of the two inference methods agreed well with each other, though the bootstrap support of maximum likelihood estimate was much lower than the posterior probabilities of Bayesian inference. Phylogenetic tree of maximum likelihood (ML) estimate and Bayesian inference showed the diversity of the chicken bHLH family (Table 1).

### 3.3. Genomic Contigs and Chromosome Locations of Chicken bHLH Genes

**Figure 2:**
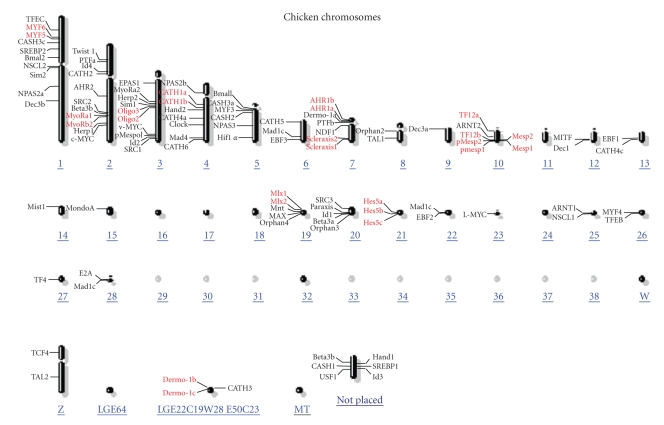
Chromosomal locations of chicken bHLH transcription factor genes. The chicken bHLH names in red are those of the same family cluster together. Family information of each bHLH gene is listed in Table 1.

Protein sequence accession number and the genomic contig number for the 104 chicken bHLH proteins are all listed in Table 1. Chromosome locations of all chicken bHLH genes are shown in Figure 2. It can be seen that chicken bHLH genes are distributed in a rather uneven pattern. While chromosomes 1, 2, 3, 4, 5, 7, 10, 19, and 20 encode 68 bHLH proteins, the remaining 33 chromosomes encode only 36 bHLH members. It should be noted that two or three chicken bHLH members that belong to the same family are found to cluster on the chromosome (Figure 2, name in red). A total of 25 chicken bHLH members fall into this category. For example, Myf5 and Myf6 cluster on chromosome 1; MyoRa1 and MyoRb2 cluster on chromosome 2; Oligo2 and Oligo3 cluster on chromosome 3; Hes5a, Hes5b, and Hes5c cluster on chromosome 21. Similar cluster patterns could also be found in human [5], rat [10], mouse [8], and zebrafish [11] genomes. This distribution pattern suggests that these bHLH members should have arisen through gene duplication at an early date, at least before the divergence of vertebrate and invertebrate species.

### 3.4. Comparison and Analysis of the bHLH Genes in Vertebrate and Invertebrate Species

**Figure 3:**
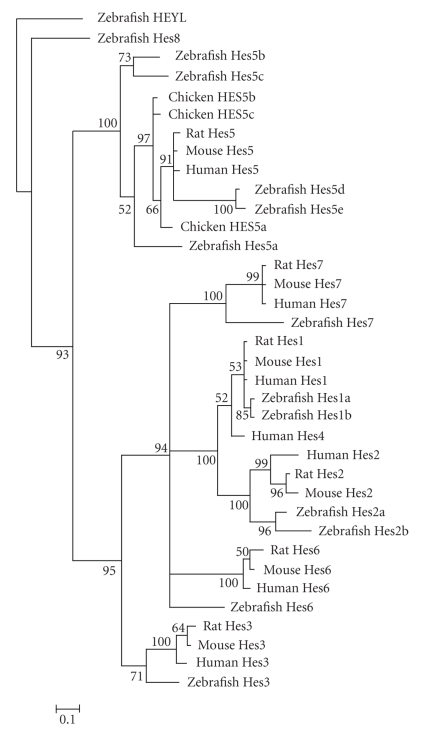
Phylogenetic tree of Hes homologues (hairy and enhancer of split) from human, mouse, rat, zebrafish, and chicken. A phylogenetic tree of Bayesian inference tree is shown. The zebrafish Heyl (hey-like) sequence was defined as the out-group. Figures around the node are Bayesian posterior probabilities of the corresponding branches. The Bayesian posterior probabilities were converted into percentages. The phylogenetic tree of Hes factor motifs revealed that Hes1, Hes2, Hes3, Hes5, Hes6, and Hes7 had their own common ancestor sequences, respectively.

A comparison of bHLH members in vertebrate and invertebrate species was made across four vertebrate and three invertebrate species (Table 2). Vertebrates have more than half the number of bHLH members that invertebrates have, and many families in vertebrates have more members, such as E12/E47, NeuroD, Atonal, Mesp, Twist, Paraxis, SCL, SRC, Myc, Mad, MITF, HIF, Emc, Hey, Coe, and other families. Among the 45 bHLH families, only 10 families have a single member in zebrafish, chicken, rat, and mouse, respectively, while 33 and 24 families have a single member in lancelet and giant owl limpet (Table 2). It is also seen that the Delilah family is missing in vertebrate species and giant owl limpet,but exists in *Drosophila* and Lancelet. It could be attributed to the gene birth-and-death process [23] of the bHLH family evolution in vertebrate and invertebrate species. A common multicopy unit is the H/E(spl) family, especially the hairy/enhancer of split factors. In the four invertebrate species, there have either 11 or 12 members, while the vertebrate species have 6, 8, and 15 members in the H/E(spl) family. An example for the phylogenetic relationship of Hes homologues from human, mouse, rat, zebrafish, and chicken was explored. A phylogenetic tree of Bayesian inference on the hairy/enhancer of split factors (symbol Hes) homologues was constructed for the analysis of evolutionary relationships among these five vertebrate species. The zebrafish HEYL was used as the out-group. It was found that all the Hes members from human, mouse, rat, zebrafish, and chicken form clear monophyletic groups, indicating that each Hes member (except Hes4 and Hes8) has its own ancestral sequence (Figure 3), similar to what Zheng et al. found in rat and mouse [11]. This phylogenetic tree may be further used to explore the birth-and-death of gene evolution in vertebrate and invertebrate species. However, there are few bHLH members clearly defined now in invertebrates other than *Drosophila* that show clear correspondence to vertebrate genes. Further effort will need to be made in the comparison and identification of corresponding bHLH paralogs and orthologs.

### 3.5. GO Enrichment Analysis of the Chicken bHLH Protein Family

To gain a better functional understanding of the bHLH family in chicken, we collected GO enrichment data on the 104 chicken bHLH proteins with significant hyper-geometric *P* values. We identified GO terms or annotations for 83 chicken bHLH genes, including 418 associated with cellular components, 1013 with molecular functions, and 2585 for general biological processes. GO statistics analyzed with a brief summary of biological process subtypes describing each group are listed in Supplemental Table 2.

**Figure 4:**
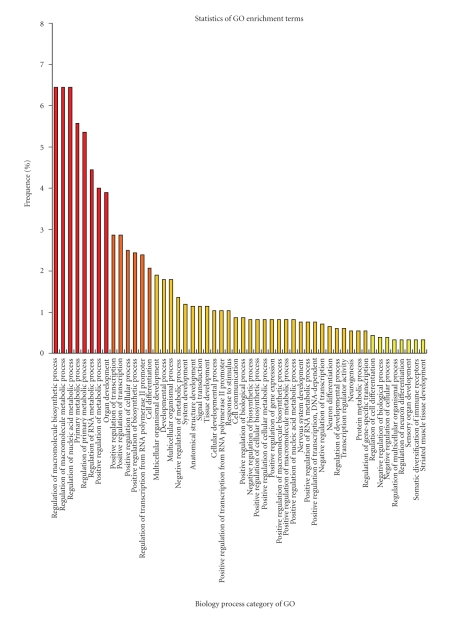
The top 51 GO terms frequency counts for chicken biological process. The bar plot indicates the numbers or frequencies of Gene Ontology (GO) terms we collected for a set of 89 biological process categories on the chicken bHLH proteins [15]. The top 51 GO annotation numbers counted more less than five were shown. Ambiguous GO terms of biology process subtypes, such as regulation of transcription, regulation of biological process, regulation of cellular process were excluded.

Our analysis focused on the collected categorical terms for 89 biological processes (BP) [15] spanning the 104 chicken bHLH proteins. The figure only shows the top 51 GO terms with frequencies of no less than ten (Figure 4). We found that when ambiguous GO categories of transcriptional factors such as the regulation of transcription, or biological or cellular processes are discounted, signal transduction, neurogenesis and neuronal differentiation, cell differentiation, and tissue development, including various regulators of biosynthetic processes and metabolic process and transcription regulation occur at high frequencies.

We have identified a near complete set of 104 chicken bHLH domains and their protein sequences in the chicken genome. Among these bHLH members, 29 hypothetical proteins such as LOC768612 (protein accession ID XP_001231238.1) were annotated, including 7 function undefined and name unknown sequences and 22 vague sequences (read as “similar to”) predicted by automated computational analysis. These uncharacterized putative bHLH proteins may be novel transcription factors, which need further validation. The basic helix-loop-helix structures of all the 29 predicted proteins have been verified by EST searching (Supplemental Table 1).

## 4. Conclusions

By TBLASTN and BLASTP searches with our 7 primer bHLH sequences of chicken and the 45 representative bHLH domains as query sequences, we identified and analyzed 104 bHLH proteins from the chicken (*Gallus. gallus*) genome and protein databases, among which 29 novel bHLH members are predicted proteins recorded in Genbank. Phylogenetic analysis of the GgbHLH domains with 118 human bHLH domains [5], we divided the chicken bHLH family into 42 subfamilies according to the 118 known human bHLH families [5, 9]. Three families, Delilah, Fig*α*, and AP4, were not found in this study. 

Chromosome distribution patterns and phylogenetic analyses strongly suggest that the bHLH members should have arisen through gene duplication at an early date, at least before the divergence of vertebrates and invertebrates. A considerable number of bHLH genes were found to have a multimember distribution pattern in human, mouse, rat, zebrafish, and chicken bHLH families, suggesting that they arose through gene duplication. Phylogenetic analysis revealed that gene duplication events should have occurred at least before the divergence of vertebrates from invertebrates. However, it still needs further effort in the comparison and identification of corresponding bHLH proteins in vertebrate and invertebrate species to explore fully the birth-and-death evolution process of bHLH transcription factors due to few clearly defined bHLH members in invertebrates other than *Drosophila* that show clear correspondence to vertebrate genes.

A primary Gene ontology (GO) analysis of the chicken bHLH transcription factor family suggested that there are much functional information enrichment in each group and different groups tend to have some certain functions. Beside of various kinds of regulation of biosynthetic process, metabolic process, gene expression and transcription regulation in cell differentiation and tissue development, signal transduction, neurogenesis and neuron differentiation have high frequencies too. It deepens our understanding of the chicken bHLH transcription factor family and provides much useful information for further studies using chicken as a model system.

## Figures and Tables

**Table 1:** A complete list of 104 bHLH genes from chicken (*Gallus gallus*) with the corresponding human homologue information.

Group	Family	Gallus gallus	Protein ID (GenBank Accession number)	Homo sapiens	BI posterior probability (%)^a^	ML Bootstrap value (%)^b^	Genome contig link
A	ASCa	*CASH1*	NP_989743.1	*Hash1*	83	71	NW_001471698.1
A	ASCa	*CASH2*	NP_990280.1	*Hash2*	93	n/m*	NW_001471698.1
A	ASCb	*CASH3a*	XP_001232099.1 (ASCL3 transcript variant 1); XP_420985.2 (ASCL3 transcript variant 2)	*Hash3a*	100	89	NW_001471698.1
A	ASCb	*CASH3c*	XP_425485.1	*Hash3c*	51	89	NW_001471513.1
A	MyoD	*MYF3*	NP_989545.1	*MYF3*	88	95	NW_001471698.1
A	MyoD	*MYF4*	NP_989515.1	*MYF4*	100	94	NW_001471608.1
A	MyoD	*MYF5*	NP_001025534.1	*MYF5*	75	96	NW_001471512.1
A	MyoD	*MYF6*	NP_001025917.1	*MYF6*	93	99	NW_001471512.1
A	E12/E47	*TF12a*	NP_990706.2	*TF12*	54	78	NW_001471425.1
A	E12/E47	*TF12b*	hmm39106	*TF12*	54	78	NW_001471425.1
A	E12/E47	*E2A*	hmm9164	*E2A*	96	98	NW_001471627.1
A	E12/E47	*TCF3*	NP_989817.2	*TCF3*	98	97	NW_001471627.1
A	E12/E47	*TCF4*	Q90683.1	*TCF4*	55	n/m*	NW_001488824.1
A	Ngn	*CATH4a*	NP_990127.1	*HATH4a*	99	94	NW_001471685.1
A	Ngn	*CATH4c*	NP_990214.1	*HATH4c*	100	90	NW_001471449.1
A	NeuroD	*NDF1*	NP_990251.1	*NDF1*	55	n/m*	NW_001471729.1
A	NeuroD	*CATH2*	XP_418852.1	*HATH2*	97	89	NW_001471633.1
A	NeuroD	*CATH3*	NP_990407.1	*HATH3*	99	94	NW_001471747.1
A	Atonal	*CATH1a*	hmm54472	*HATH1*	100	87	NW_001471683.1
A	Atonal	*CATH1b*	XR_026796.1	*HATH1*	100	87	NW_001471683.1
A	Atonal	*CATH5*	NP_989999.1	*HATH5*	99	91	NW_001471715.1
A	Mist	*Mist1*	XP_425228.1	*Mist1*	100	98	NW_001471454.1
A	Beta3	*Beta3a*	NP_989835.1	*Beta3a*	57	62	NW_001471567.1
A	Beta3	*Beta3b*	NP_989834.1	*Beta3b*	95	76	NW_001471646.1
A	Oligo	*Oligo2*	NP_001026697.1	*Oligo2*	67	62	NW_001471669.1
A	Oligo	*Oligo3*	XP_001232806.1	*Oligo3*	84	76	NW_001471669.1
A	Net	*CATH6*	XP_001234980.1	*HATH6*	96	98	NW_001471687.1
A	Mesp	*Mesp1*	hmm11657	*Mesp1*	n/m	n/m	NW_001471429.1
				*Mesp2*			
A	Mesp	*Mesp2*	NP_989897.1	*Mesp1*	n/m	n/m	NW_001471429.1
				*Mesp2*			
A	Mesp	*pMesp1*	hmm17962	*pMesp1*	n/m	n/m	NW_001471429.1
				*pMesp2*			
A	Mesp	*pMesp2*	XP_001231219.1	*pMesp1*	n/m	n/m	NW_001471429.1
				*pMesp2*			
A	Mesp	*pMespo1*	NP_990015.1	*pMesp1*	n/m	n/m	NW_001471673.1
				*pMesp2*			
A	Twist	*Twist1*	NP_990070.1	*Twist1*	96	82	NW_001471633.1
A	Twist	*Dermo-1a*	NP_990010.1	*Twist2*	98	92	NW_001471728.1
A	Twist	*Dermo-1b*	NP_001096684.1	*Twist2*	100	98	NW_001471747.1
A	Twist	*Dermo-1c*	XP_424492.1	*Twist2*	100	98	NW_001471747.1
A	Paraxis	*Paraxis*	NP_990277.1	*Paraxis*	79	74	NW_001471567.1
A	Paraxis	*Scleraxis1*	NP_989584.1	*Scleraxis*	95	92	NW_001471733.1
A	Paraxis	*Scleraxis2*	XP_001234790.1	*Scleraxis*	91	97	NW_001471733.1
A	MyoRa	*MyoRa1*	XP_418293.2	*MyoRa1*	80	79	NW_001471650.1
A	MyoRa	*MyoRa2*	XP_419734.1	*MyoRa2*	100	n/m*	NW_001471669.1
A	MyoRb	*MyoRb2*	XP_427081.2	*MyoRb2*	85	n/m*	NW_001471649.1
A	Hand	*Hand1*	NP_990296.1	*Hand1*	99	91	NW_001471449.1
A	Hand	*Hand2*	NP_990297.1	*Hand2*	100	98	NW_001471685.1
A	PTFa	*PTFa*	XP_425989.1	*PTFa*	100	98	NW_001471633.1
A	PTFb	*PTFb*	XP_001234487.1	*PTFb*	99	95	NW_001471728.1
A	SCL	*TAL1*	NP_990683.1	*TAL1*	60	62	NW_001471740.1
A	SCL	*TAL2*	XP_424886.1	*TAL2*	99	82	NW_001488876.1
A	NSCL	*NSCL1*	NP_989452.1	*NSCL1*	100	99	NW_001471598.1
A	NSCL	*NSCL2*	NP_990128.1	*NSCL2*	72	85	NW_001471526.1
B	SRC	*SRC1*	NP_001012900.1	*SRC1*	91	98	NW_001471673.1
B	SRC	*SRC2*	XP_001231617.1	*SRC2*	100	98	NW_001471649.1
B	SRC	*SRC3*	XP_417385.2	*SRC3*	99	86	NW_001471567
B	MYC	*v-MYC*	NP_001026262.1	*v-MYC*	100	89	NW_001471673.1
B	MYC	*c-MYC*	NP_001026123.1	*c-MYC*	100	56	NW_001471654.1
B	MYC	*L-MYC*	XP_425790.1	*L-MYC1, L-MYC2*	98	98	NW_001471589.1
B	Mad	*Mad1a*	NP_001034399.1	*Mad1 (Mxi1)*	98	96	NW_001471581.1
B	Mad	*Mad1c*	NP_001012929.1	*Mad1 (Mxi1)*	98	74	NW_001471720.1
B	Mad	*Mad4*	NP_001006460.1	*Mad4*	100	85	NW_001471687.1
B	Mnt	*Mnt*	XP_425414.2	*Mnt*	98	68	NW_001471508.1
B	MAX	*MAX*	P52162.1	*MAX*	100	91	NW_001471508.1
B	USF	*USF1*	NP_001007486.1	*USF1*	92	82	NW_001474499.1
B	MITF	*MITF*	NP_990360.1	*MITF*	100	64	NW_001471443.1
B	MITF	*TFEB*	NP_001026093.1	*TFEB*	100	96	NW_001471610.1
B	MITF	*TFEC*	NP_001006229.1	*TFEC*	100	71	NW_001471512.1
B	SREBP1	*SREBP1*	NP_989457.1	*SREBP1*	100	96	NW_001471454.1
B	SREBP2	*SREBP2*	XP_416222.2	*SREBP2*	100	99	NW_001471513.1
B	Mlx	*Mlx1*	NP_001104311.1	*Mlx*	96	n/m*	NW_001471508.1
B	Mlx	*Mlx2*	hmm20496	*Mlx*	96	n/m*	NW_001471508.1
B	Mlx	*MondoA*	hmm54830	*MondoA*	100	91	NW_001471459.1
B	TF4	*TF4*	NP_001026101.1	*TF4*	100	83	NW_001471622.1
C	Clock	*Clock*	NP_989505.2	*Clock*	98	87	NW_001471686.1
C	Clock	*NPAS2a*	NP_001025713.1	*NPAS2*	100	97	NW_001471545.1
C	Clock	*NPAS2b*	XP_420353.2	*NPAS2*	100	99	NW_001471681.1
C	ARNT	*ARNT1*	NP_989531.1	*ARNT1*	100	100	NW_001471606.1
C	ARNT	*ARNT2*	XP_413854.2	*ARNT2*	100	100	NW_001471428.1
C	Bmal	*Bmal1*	NP_001001463.1	*Bmal1*	71	85	NW_001471698.1
C	Bmal	*Bmal2*	NP_989464.1	*Bmal2*	100	n/m*	NW_001471513.1
C	AHR	*AHR1a*	hmm34307	*AHR1*	68	94	NW_001471728.1
C	AHR	*AHR1b*	hmm34113	*AHR1*	68	94	NW_001471728.1
C	AHR	*AHR2*	hmm46108	*AHR2*	70	90	NW_001471639.1
C	Sim	*Sim1*	XP_419817.2	*Sim1*	74	n/m*	NW_001471671.1
C	Sim	*Sim2*	XP_416724.2	*Sim2*	93	88	NW_001471534.1
C	Trh	*NPAS3*	XP_421232.2	*NPAS3*	73	n/m*	NW_001471710.1
C	HIF	*Hif1*α**	NP_989628.1	*Hif1*α**	100	92	NW_001471710.1
C	HIF	*EPAS1*	NP_990138.1	*EPAS1*	100	91	NW_001471679.1
D	Emc	*Id1*	NP_989921.1	*Id1*	69	n/m*	NW_001471567.1
D	Emc	*Id2*	NP_990333.1	*Id2*	98	89	NW_001471673.1
D	Emc	*Id3*	NP_989920.1	*Id3*	100	96	No clear
D	Emc	*Id4*	NP_989613.1	*Id4*	91	86	NW_001471637.1
E	Hey	*Herp1*	XP_425926.2	*Herp1*	97	89	NW_001471651.1
E	Hey	*Herp2*	XP_419754.2	*Herp2*	66	73	NW_001471671.1
E	H/E(spl)	*Dec1*	hmm32419	*Dec1*	82	80	NW_001471443.1
E	H/E(spl)	*Dec3a*	XP_422641.2	*?*	n/m	n/m	NW_001471743.1
E	H/E(spl)	*Dec3b*	XP_416543.2	*?*	n/m	n/m	NW_001471526.1
E	H/E(spl)	*Hes5a*	NP_001012713.1	*Hes5*	75	78	NW_001471571.1
E	H/E(spl)	*Hes5b*	XP_417552.2	*Hes5*	n/m	97	NW_001471571.1
E	H/E(spl)	*Hes5c*	XP_417553.2	*Hes5*	n/m	97	NW_001471571.1
F	Coe	*EBF1*	NP_990083.1	*EBF1*	52	n/m*	NW_001471449.1
F	Coe	*EBF2*	XP_417675.2	*EBF2*	94	90	NW_001471575.1
F	Coe	*EBF3*	XP_421824.2	*EBF3*	67	n/m*	NW_001471723.1
?	Orphan	*Orphan2*	XP_422318.1	*?*	n/m	n/m	NW_001471740.1
?	Orphan	*Orphan3*	XP_001234727.1	*Orphan3*	100	93	NW_001471567.1
?	Orphan	*Orphan4*	XP_001235101.1	*?*	n/m	n/m	NW_001471508.1

Chicken bHLH genes were named according to their human homologues. Bootstrap values were from phylogenetic analyses with human bHLH sequences using Bayesian inference and ML algorithm, respectively. BI posterior probability (note a) refers the result from Bayesian inference in phylogenetic analysis, and ML bootstrap value (note b) refers the result from maximum likelihood estimate in phylogenetic analysis. The numbers in the phylogenetic trees are converted into percentages. All bHLH members are in the order of bHLH families manifested in Ledent et al. [5, Table 1]. All protein sequences were retrieved in NCBI website except those numbered beginning with “hmm” which were from database of “*Ab initio* protein”. The question mark means no matching, mark n/m means none monophyletic group with another single bHLH sequence of a known family, but formed a monophyletic group with two or more homologue sequences of the same family; n/m* denotes cases of lower bootstrap value estimated less than 50%.

**Table 2:** A comparison of the number of bHLH factors among vertebrate and invertebrate species.

Family	Group	*Drosophila*	Lancelet	Giant owl limpet	Chicken	Zebrafish	Rat	Mouse
ASCa	A	4	3	6	2	2	2	2
ASCb	A	0	1	1	2	3	3	3
MyoD	A	1	4	1	4	4	4	4
E12/E47	A	1	1	4	5	5	4	4
Ngn	A	1	1	3	2	2	3	3
NeuroD	A	0	1	1	3	5	4	4
Atonal	A	3	1	2	3	4	2	2
Mist	A	1	1	1	1	1	1	1
Beta3	A	1	1	2	2	3	2	2
Oligo	A	0	2	3	2	4	3	3
Net	A	1	1	2	1	1	1	1
Delilah	A	1	1	0	0	0	0	0
Mesp	A	1	1	0	5	5	3	3
Twist	A	1	1	2	4	3	2	2
Paraxis	A	1	2	1	3	4	2	2
MyoRa	A	1	4	1	2	2	2	2
MyoRb	A	0	1	1	1	2	2	2
Hand	A	1	1	1	2	1	2	2
PTFa	A	1	1	1	1	1	1	1
PTFb	A	2	3	1	1	2	1	1
SCL	A	1	1	5	2	3	3	3
NSCL	A	1	1	1	2	1	2	2
SRC	B	1	1	0	3	3	3	3
Fig*α*	B	0	1	0	0	1	1	1
Myc	B	1	1	1	3	6	4	4
Mad	B	0	1	1	3	4	4	4
Mnt	B	1	1	1	1	2	1	1
Max	B	1	1	1	1	1	1	1
USF	B	1	1	2	1	2	2	2
MITF	B	1	1	1	3	5	4	4
SREBP	B	1	1	1	2	2	2	2
AP4	B	1	1	1	0	1	1	1
MLX	B	1	1	7	3	1	2	2
TF4	B	1	0	1	1	1	1	1
Clock	C	3	1	2	3	3	2	2
ARNT	C	1	1	0	2	2	2	2
Bmal	C	1	1	0	2	2	2	2
AHR	C	2	1	1	3	4	2	2
Sim	C	1	1	1	2	2	2	2
Trh	C	1	1	0	1	2	1	1
HIF	C	1	1	1	2	6	4	4
Emc	D	1	1	2	4	5	4	4
Hey	E	1	1	1	2	4	4	4
H/E(spl)	E	11	11	12	6	15	8	8
Coe	F	1	1	1	3	5	4	4
Orphan	?	0	6	4	3	2	4	4

Total		59	78	82	104	139	114	114

The vertebrate and invertebrate species referred lancelet (B*ranchiostoma floridae*), giant owl limpet (*Lottia gigantean*), *Drosophila* (*Drosophila melanogaster*, fruit fly), zebrafish (*Danio rerio*), chicken (*Gallus gallus*), rat (*Rattus norvegicus*), and mouse (*Mus musculus*). Data on lancelet and *Drosophila* are from Simionato et al. [9]. Data on zebrafish, rat, and mouse are from Wang et al. [10] and Zheng et al. [11]. Data on giant owl limpet and chicken are from the findings of this study. Family names and group assignment followed Ledent et al. [5, Table 1].

## Abbreviations

Hs: *Homo* *s* *a* *p* *i* *e* *n* *s*
Gg: *Gallus* *gallus*.
